# Complete Genome Sequences of Two Marine *Vibrio cholerae* Strains Isolated from the South Coast of Sweden

**DOI:** 10.1128/genomeA.01118-16

**Published:** 2016-10-27

**Authors:** Kaisa Thorell, Betty Collin, Bodil Hernroth, Åsa Sjöling

**Affiliations:** a Department of Microbiology, Tumor and Cell Biology, Karolinska Institutet, Stockholm, Sweden; b Department of Natural Science, Kristianstad University, Kristianstad, Sweden; c The Royal Swedish Academy of Sciences, Sven Lovén Centre-Kristineberg, Fiskebäckskil, Sweden

## Abstract

*Vibrio cholerae* serogroups O1 and O139 are commonly associated with diarrhea, while non-O1-O139 strains may cause wound infections. Here, we present the genome sequences of two *V. cholerae* strains isolated from blue mussels (*Mytilus edulis*) collected in coastal waters of southern Sweden.

## GENOME ANNOUNCEMENT

*Vibrio cholerae* is a Gram-negative aquatic bacterium, and cholera toxin (*ctxAB*)-producing strains may cause cholera (1). The great majority of marine *V. cholerae* strains are apathogenic and do not carry any biomarkers commonly used in virulence screening. However, these *Vibrio* species may cause wound infections and septicemia, and two fatal cases were recorded in Sweden during the summer of 2006. No biomarkers have yet been detected to identify these strains (2, 3).

The sequenced strains (L11 and L15) were isolated from blue mussels (*Mytilus edulis*) collected in Lomma Bay in southern Sweden in 2007. Initial molecular studies showed that the strains did not carry standard virulent biomarkers (i.e., *ctxA, tcpA*, and *zot*) (4). A previous study conducted on L11 revealed that this strain was resistant against killing by mussel hemocytes and harbored the type 3 secretion system (T3SS), indicating virulence (5). When inoculated in a sterile cold-water (4°C) microcosm, L11 remained culturable for at least three weeks (B. Collin and B. Hernroth, unpublished data). Increased knowledge about the gene diversity of environmental strains is necessary to explore biomarkers of interest for the health of both humans and shellfish.

The isolates were grown at 37°C overnight, and the genomic DNAs were extracted by using the blood and tissue kit (Qiagen, Valencia, CA, USA), according to the manufacturer’s manual.

Sequencing libraries were prepared using the TruSeq Nano DNA library prep (Illumina, San Diego, CA, USA). The libraries were sequenced at the Genomics Core Facility, Sahlgrenska Academy, Gothenburg, Sweden, on the Illumina MiSeq instrument with 2 × 250-bp paired-end reads reaching >3.5 million reads for L11 and 2.3 million reads for L15. The reads were quality controlled and filtered with Trim Galore! (http://www.bioinformatics.babraham.ac.uk/projects/trim_galore/), using a cutoff of Q30, and assembled *de novo* using SPAdes version 3.8.0 (6). After filtering away contigs <500 bp and contigs of very low coverage, the draft genomes were ordered against the complete non-O1 *Vibrio cholerae* LMA3894-4 genome (accession numbers CP002555.1 and CP002556.1), using Mauve (7) and annotated with the Prokka pipeline (8), using *V. cholerae* N16961 (RefSeq accession numbers NC_002505.1 and NC_002506.1) as the primary annotation source.

The draft genome of strain L11 consists of 30 contigs and has a size of 4,000,957 bp. The G+C content is 47.5%, and 3,622 coding sequences, 64 tRNA genes, and seven rRNA genes were identified with Prokka. The draft genome of strain L15 has a size of 4,083,945 bp and a G+C content of 47.4%, and 3,576 coding sequences, 81 tRNA genes, and eight rRNA genes were identified with Prokka.

Using the Roary software for pangenome analysis (9), with an identity cutoff of 80%, we found that L11 and L15 shared 3,122 genes, while L11 had 437 unique genes and L15 had 416 unique genes. L15 carried two putative antibiotic resistance genes: the phenicol resistance gene VCA0300 (chloramphenicol acetyltransferase), and the beta-lactam resistance gene CARB-6 precursor.

A more detailed comparison of these strains will be presented in future publications.

### Accession number(s).

The whole-genome shotgun projects have been deposited at DDBJ/ENA/GenBank under the accession numbers MDYI00000000 (*V. cholerae* L11) and MDYJ00000000 (*V. cholerae* L15). The versions described in this paper are versions MDYI01000000 and MDYJ01000000, respectively.
